# Gene Network Revealed Involvements of Birc2, Birc3 and Tnfrsf1a in Anti-Apoptosis of Injured Peripheral Nerves

**DOI:** 10.1371/journal.pone.0043436

**Published:** 2012-09-17

**Authors:** Yongjun Wang, Xin Tang, Bin Yu, Yun Gu, Ying Yuan, Dengbing Yao, Fei Ding, Xiaosong Gu

**Affiliations:** Key Laboratory of Neuroregeneration, Nantong University, Nantong, PR China; Catholic University Medical School, Italy

## Abstract

Crush injury or axotomy of peripheral nerves results in the rapid production of the inflammatory cytokines, which were confirmed in various models, to some extent, to be noxious to the myelin sheath or Schwann cells (SCs). TNF-α is one of the primary initiators of the inflammatory cascade and exerts pleiotropic functions in the physiological conditions by binding to its receptors, type I (TNFRI) and type II (TNFRII). The pathway molecules TNFRI, Birc2 and Birc3 play key roles during the activation of the signaling. Injured peripheral nerves, preventing them from TNF-α-mediated destruction and proceeding to successful regeneration, might initiate an anti-apoptotic mechanism. To identity the exact functions of TNFRI, Birc2 and Birc3, as well as its involved pathways in the cellular events, we inferred a dynamic gene regulatory network from short time-series measurements of the proximal nerve segment cDNA microarray following rat sciatic nerve transection. TNFRI family member Tnfrsf1a, Birc2 and Birc3 were mined out integrating as master regulators to mediate inflammatory responses. Experiments revealed that Tnfrsf1a, Birc2 and Birc3 proteins colocalized with S100 in the rat peripheral nerve tissues, and the expression levels increased with the time extension. Knockdown of the proteins induced the apoptotic formation of primary cultured SCs by upregulation of caspase 3 and caspase 6. Our systematic analysis indicated that Tnfrsf1a, Birc2 and Birc3 of SCs, not originally regarded as XIAP, were mainly responsible for the inflammation-mediated anti-apoptosis of peripheral nerves. Birc2 and Birc3 might be the most potential targets for anti-apoptotic protection mediated by inflammatory cytokines.

## Introduction

The myelin sheath performs functions in promoting rapid salutatory conduction, the maintenance of normal axonal transport and providing trophic support to axons [1]. Dysmyelination or demyelination of axons often contributes to the abnormalities in calibre, axonal transport, the phosphorylation and packing of neurofilaments, and the organization of ion channels in the axonal membrane [2]. Such cases have been typically observed in demyelinating disorders of both central nervous system (CNS) and peripheral nervous system (PNS), as well as in genetic diseases that lead to abnormal myelination [2]–[5]. Extensive investigations have clearly revealed that multiple intrinsic and extrinsic factors are implicated in dysmyelination or demyelination, including genetic defects, toxins, metabolic disturbances, infections, inflammation, ischaemia, paraneoplastic disorders, and physical injuries [3], [6]–[10]. Among which, inflammatory factors have been highlighted for their pivotal roles in orchestrating immune and inflammatory cell-cell interactions and representing potentially noxious molecules to the myelin sheath or Schwann cells (SCs) [11], [12]. An example revealed that TNF-α inhibition with soluble receptors or neutralizing antibodies could reduce inflammatory demyelination in various animal models of neuropathy [13], suggesting a close relation of the cytokine to demyelination.

Crush injury or axotomy of PNS was found to trigger the rapid production of the inflammatory cytokines, which contributed to macrophage recruitment [14]–[16]. These recruited macrophages may further release and result in an increase in both kinds and amounts of inflammatory cytokines, thus directly or indirectly regulate molecular and cellular events of injured peripheral nerves. Anti-inflammatory cytokines and other molecules responsible for turning off pro-inflammatory cytokines, however, decreased at early stages and increased at 7 days after injury [14]. Thus, several types of cells which rapidly arrived, might initiate a self-protective mechanism against the challenges of excessive inflammatory cytokines, such as cross-tolerization of macrophages to TNF-α [17]. In some occasions, SCs might select functional activation of NFκB and c-jun pathways for anti-apoptosis [18], [19]. How the proximal stump prevents it from the damages of inflammatory cytokines, and maintains the structural and functional integrity except a retrograde degeneration in a short segment [20], is still unclear. Understanding the molecular basis of the proximal nerve anti-inflammation would open the possibility of targeting relative signals as therapeutic interventions for inflammation-mediated demyelination.

TNF-α is thought to be one of the primary initiators of the inflammatory cascade, and to act as potent chemoattractants for macrophages and other white blood cells [21], [22]. In axotomised peripheral nerves, TNF-α is capable of inducing the reversion of mature to immature SCs following the decrease in axonally derived trophic support. This phenotype reversion, with increased p75^NTR^ expression, increases the cell's susceptibility to pro-apoptotic molecular mechanisms [12]. It is widely accepted that this pleiotropic cytokine exerts its functions by binding to its receptors, type I (TNFRI) and type II (TNFRII). Both receptors are independently able to activate the pro-survival NFκB signaling pathways. However, TNFRI has the potential to induce apoptosis by activation of initiator caspase-8 and then effectors caspase 3, caspase 6, caspase 7 [23]. Birc2 and Birc3 (originally known as inhibitor of apoptosis protein cIAP1 and cIAP2) play important roles in the regulation of TNF-α pro-apoptotic signaling. They not only suppress TNF-α stimulated cell apoptosis by preventing formation of the TNFR1 pro-apoptotic signaling complex, but also regulate pro-survival NFκB signaling pathways in the non-canonical pathway by ubiquitination of NFκB-inducing kinase (NIK), and in the canonical pathway by a yet-to-be-defined mechanism [24], [25]. We herein postulate that the proximal nerves is likely to produce anti-apoptotic response against the TNF-α-mediated inflammation, and the pathway molecules Birc2, Birc3 and TNFRI might correspondingly play key roles during the activation of the signaling. In the present study, we inferred the signaling networks by integrating gene expression profiling data derived from the proximal stump of the sciatic nerve following injury, using bioinformatic approaches, in attempt to identify major gene regulators Birc2, Birc3 and TNFRI. By understanding how the pathways fit together, we can combine our knowledge of mechanisms for anti-inflammatory protection.

## Results

### Temporal expression profiles and enriched GO terms of injury-related genes in proximal nerve stump

The cDNA microarray technique was adapted to analyze the temporal expression profiles of both proximal and distal segments at various times following sciatic nerve axotomy. We selected short time points due to the rapid production of inflammatory cytokines [16], and TNF-α has been found to modulate peripheral nerve development and function via SCs-axon interactions within several hours in the DRG coculture system [26]. The underlying signaling mechanism involved in the process is unclear. Furthermore, there were no significant functional annotations of TNF-α-related signals identified in the long time gene networks deduced from sciatic nerve injury (Data not shown). As such, TNF-α-mediated anti-apoptosis or apoptosis is possibly predicted to occur in the short time points. A total of 2850 differentially expressed genes were identified from proximal nerve segments at 6 time-points after sciatic nerve transection, by adjusting P-value less than 0.01 and controlling for a 5% FDR. SCs are the prevalent cell type in the myelinated axon [27], suggesting that the genes analyzed were mostly extracted from the SCs. Initial analysis based on signal density changes revealed that the expression profiles of these genes were categorized into 20 models of STC in the time series (ANOVA, P<0.05) (Data not shown). As proteins participating in similar biological pathways often have similar expression profiles [28], we attempted to gain interesting functional insights from the temporal expression profiles with the lowest P-value. Profiles of 3 models with minimum P-values, which contained 453, 233 and 224 differentially expressed genes, respectively, were thus selected for GO terms analysis [29].

The five most significant GO terms for each model revealed that functional annotations of signal initiation, regulation of signaling process, response to external stimulus, regulation of cell communication and cytokine production were found in the first model. While annotations of external stimulus, cell activation, inflammatory response, cell death and chemotaxis were present in the second. Annotations of translation, protein metabolic process, protein maturation by peptide bond cleavage, negative regulation of adiponectin secretion and neutrophil chemotaxis were exhibited in the third (Data not shown).

### Identification of major gene regulators Birc2, Birc3 and Tnfrsf1a from gene network of injured peripheral nerves

**Figure 1:**
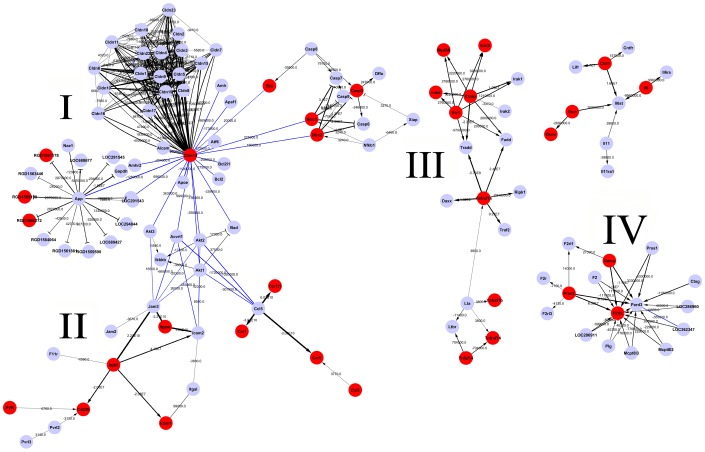
Gene network analysis of the proximal nerve segments in short time-series following rat sciatic nerve transection. The lines indicate the interactions between each gene, and the weight value is illustrated by the width of lines. The black line represents those interactions existing in KEGG database, and the blue lines represent those interactions obtained by computation.

Pathways overrepresented in the injury-related genes were searched based on the KEGG database. A total of 9 canonical pathways were identified (*Fisher*'s exact test, P-value<0.001), including cytokine-cytokine receptor interaction, cell adhesion molecules, Toll-like receptor signaling pathway and apoptosis, etc (Table 1). These signaling processes are important for neuron survival, axonal-SCs interaction and responses to inflammatory stimulus. Our goal was to construct a gene network and to identify major gene regulators Birc2, Birc3 and TNFRI, which reflect functionally significant molecular events in cellular pathways. Therefore, differentially expressed genes at six time-points were organized into an interactome network [30], based on the results of the pathway analysis. The weight value of each gene in the network was calculated, representing the effect of the gene on the downstream targets (Figure 1, Table 2). Fortunately, Birc2, Birc3 and Tnfrsf1a (tumor necrosis factor receptor superfamily member 1a) acting as gene regulators at central nodes, were traced in one of four sub-networks associated with inflammation and anti-apoptosis (Figure 1, III). These TNF-α signaling pathway members were predicted to regulate apoptosis-related factors including caspase 6, caspase 3, Daxx and Traf2, etc. In addition, the other three sub-networks involved in intercellular tight junction, cell polarity and adhesion were also excavated.

**Table 1:** KEGG pathway analysis.

Pathway	Count	*P* value	qFDR
Cytokine-cytokine receptor interaction	51	1.69E−14	1.79E−12
Hematopoietic cell lineage	19	2.96E−06	7.82E−05
Cell adhesion molecules	27	2.97E−05	0.0006
Jak-STAT signaling pathway	26	3.63E−05	0.0006
MAPK signaling pathway	38	5.32E−05	0.0008
Toll-like receptor signaling pathway	19	8.82E−05	0.001
Natural killer cell mediated cytotoxicity	19	0.0004	0.005
Glycolysis/Gluconeogenesis	11	0.0006	0.007
Apoptosis	17	0.001	0.012

**Table 2:** Effect of key regulated gene on the downstream genes.

Gene 1	Gene 2	Weight value	Interaction
Cldn14	Cldn5	7.25E+10	binding/association
Cldn14	Cldn19	4.54E+10	binding/association
Cldn14	Cldn22	3.96E+07	binding/association
Cldn14	Cldn1	1.82E+07	binding/association
Birc2	Casp6	−1.30E+07	inhibition
Birc2	Casp3	−4.27E+07	inhibition
Birc3	Casp3	−7440000	inhibition
Birc3	Casp6	−2.36E+07	inhibition
Tnfrsf1a	Daxx	4.62E+08	activation
Tnfrsf1a	Traf2	9.27E+07	activation
Il1rap	Irak1	1.09E+07	activation
Il1rap	Irak3	3220000	activation
Il1rap	Irak4	3220000	activation
Il1rap	Myd88	3220000	activation
Il1rap	Fadd	2800000	activation
Il1r1	Irak3	2760000	activation
Il1r1	Irak4	2760000	activation
Il1r1	Myd88	2760000	activation
Il1r1	Irak1	1210000	activation
Clcf1	Il6st	1.68E+07	activation
Il6	Il6ra	9960000	activation
Osm	Il6st	2690000	activation
Itgb2	Icam2	4.15E+07	activation
Prss2	F2rl2	6.08E+07	activation
Gzma	F2rl2	1.18E+07	activation

In comparison, a total of 1546 differentially expressed genes were identified in distal nerve segments collected at 0 h, 0.5 h, 1 h, 3 h, 6 h, 9 h and 12 h. By using the same approaches mentioned above, 30 canonical pathways including apoptosis were identified to be implicated in the cellular events in response to the axotomy (Fisher's exact test, P-value<0.001, Table S1). Tnfrsf1a, Myd88 and Il1r2 were found to activate apoptosis-related molecules FADD, caspase 3 or (and) Ripk1 (Figure S1, Table S2). Birc3 was shown to inhibit the activity of caspase 3 (Figure S1). Another anti-apoptotic molecule, Birc2, was not circled in the ranges of differentially expressed genes. These results suggested that the distal segments of axotomised sciatic nerve emerged both promoting and inhibiting apoptotic changes under regulation of several molecules, comparing with the anti-apoptotic changes mediated by Birc2, Birc3 and Tnfrsf1a in the proximal segments.

### TNF-α protein production in intact and injured PNS, and high dose of TNF-α induces Schwann cell apoptosis

**Figure 2:**
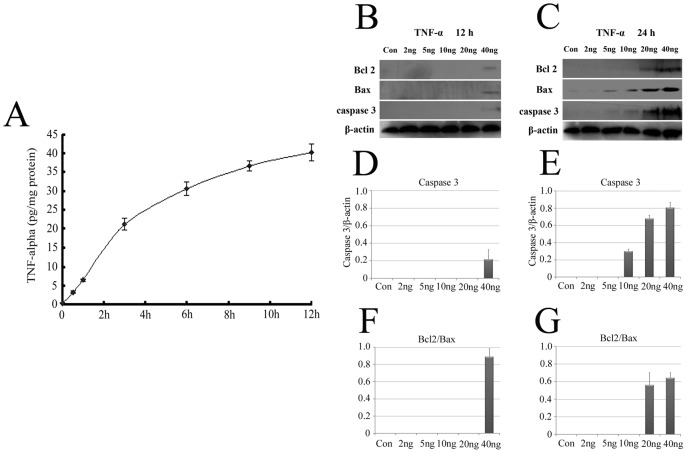
TNF-α protein production in intact and injured PNS, and its effects on SCs apoptosis. A, Demonstrated that TNF-α was detected by ELISA in the proximal nerves at 0, 0.5 h, 1 h, 3 h, 6 h, 9 h and 12 h post-transection, respectively. The effects of different concentrations of TNF-α on SCs apoptosis after treatment for 12 h (B, D and F) and 24 h (C, E and G) were determined by western blots using cleaved caspase 3, Bcl 2 and Bax antibodies. Error bars indicate ±1 SEM.

TNF-α signaling pathway was triggered and acted roles following sciatic nerve transection, implying an association with the production of TNF-α protein. We therefore tested for the production of the inflammatory cytokine by ELISA in extracts of proximal segments. The content of TNF-α was 0.23 pg per mg of total proteins in intact PNS segments, and increased with the time extension after injury. TNF-α content was 3.2 pg, 6.5 pg, 21.2 pg, 30.6 pg, 36.7 pg and 40.2 pg per mg of total proteins in injured PNS segments following transection for 0.5 h, 1 h, 3 h, 6 h, 9 h and 12 h, respectively (Figure 2A). To further evaluate the effects that increased TNF-α exerts on SCs after axonal injury, SCs were treated with exogenous TNF-α *in vitro*, at concentration of 0–40 ng/ml for 12 h and 24 h (Figure 2B–G). The apoptosis of SCs induced by TNF-α was determined by cleaved caspase 3. This cleavage was observed in the cells treated with 40 ng/ml TNF-α for 12 h or 10–40 ng/ml for 24 h, suggesting that high dose of TNF-α induces SCs apoptosis in agreement with the previous observations [12]. It was interesting to note that Bcl2 was also detectable in response to challenges of high dose of TNF-α.

### Birc2, Birc3 and Tnfrsf1a were upregulated in proximal segments following peripheral nerve transection

**Figure 3:**
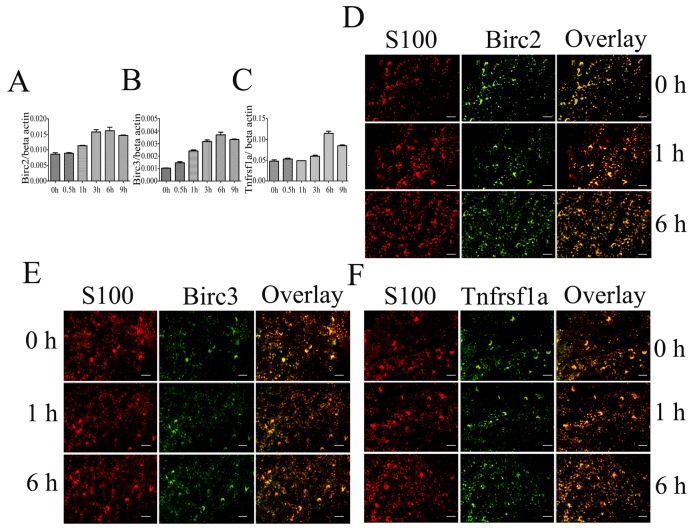
Expression analysis of major gene regulators Birc2, Birc3 and Tnfrsf1a. A–C, Quantitative real-time PCR determination of Birc2, Birc3 and Tnfrsf1a from proximal nerve segments post sciatic nerve transection at different time-points. Quantities were normalized to endogenous β-actin expression. Error bars represent standard deviation. D–F, Immunohistochemical analysis of the proximal nerve segments, showing that Birc2, Birc3 and Tnfrsf1a localized in the SCs, respectively, and that positive stainings increased at 6 h postaxatomy. Scale bars, 50 µm.

To ascertain the exact physiological functions of TNF-α pathway member Birc2, Birc3 and Tnfrsf1a in the proximal stump following peripheral nerve injury, we firstly examined both transcriptional and protein levels in peripheral nerves. qRT-PCR experiments using mRNA isolated from proximal nerve segments demonstrated that the expression of *Birc3* began to increase at 0.5 h after nerve transection, whereas expressions of *Birc2* and *Tnfrsf1a* began to increase at 1 h and 6 h, respectively (Figure 3A–C). The expression levels of the three proteins also showed an increase from 0.5 h, and peaked at 3 h or 6 h (Figure S2), similar to the transcriptional levels. Tissue immunohistochemistry revealed that all the three proteins colocalized with S100, not with NF (Data not shown), indicating that the major gene regulators playing physiological roles are temporally modulated by SCs (Figure 3D–F).

As displayed in the gene networks of distal segments, the protein of Birc2 was not detected in the experimental times by western blot, whereas both Birc3 and Tnfrsf1a were upregulated from 0.5 h, and peaked at 1 h (Figure S2). Though the cDNA microarray has screened both apoptotic and anti-apoptotic molecular changes, extensive investigation has shown that the distal segment has complex molecular regulatory mechanism leading to the SCs circumvention of apoptosis. We also found that the cleaved caspase 3 was undetectable in the distal segments (Figure S2), and TUNEL staining further confirmed that there appeared no SCs apoptosis except for the surrounding perineurium (Figure S2).

### Involvements of Birc2, Birc3 and Tnfrsf1a in anti-apoptosis of primary Schwann cells

**Figure 4:**
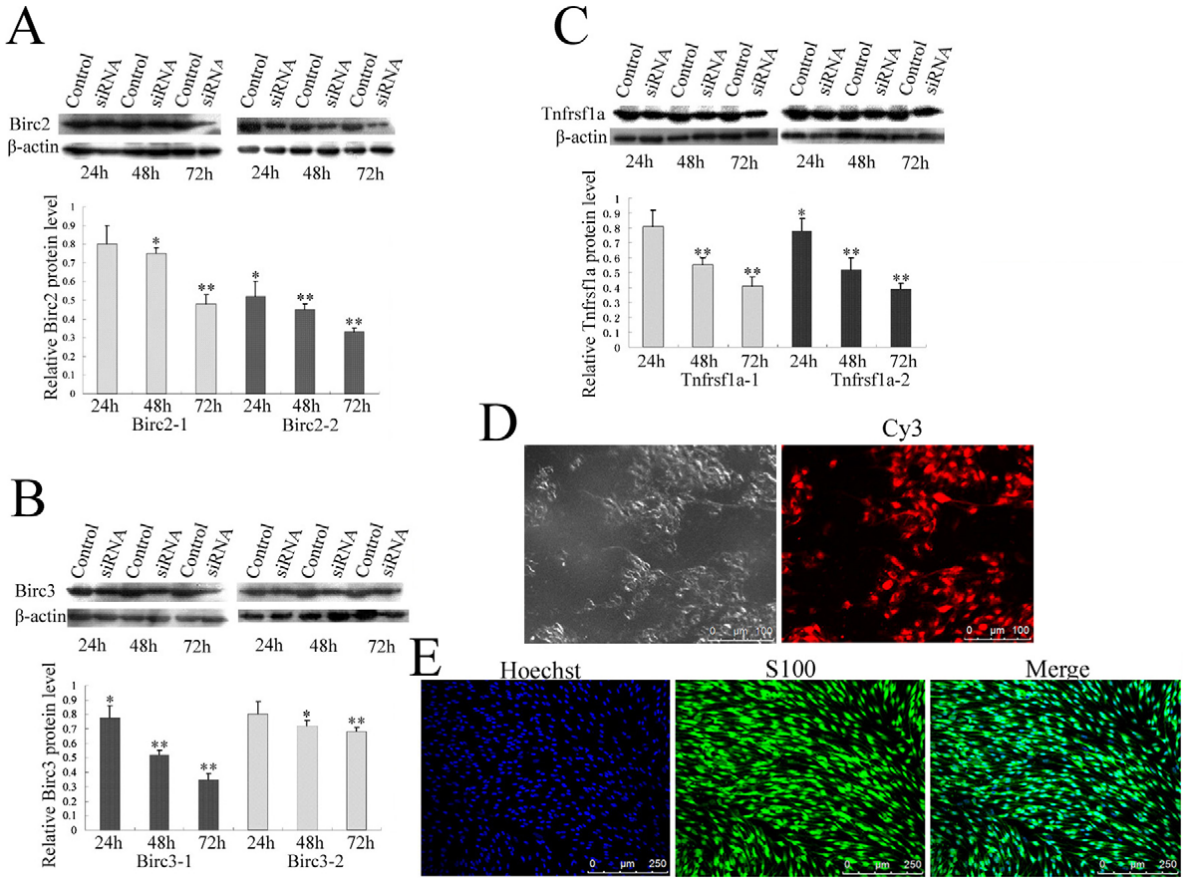
Determination of siRNA interference in SCs. A–C, Interference efficiency of two siRNA oligonucleotides for Birc2, Birc3 and Tnfrsf1a was measured by Western blot, and siRNA oligonucleotides Birc2-2, Birc3-1 and Tnfrsf1a-2 were used for further knockdown experiments, respectively; D, Determination of siRNA transfection efficiency by Cy3 control; E, Determination of SCs purity by immunoreactivity of Hoechst and S100.

**Figure 5:**
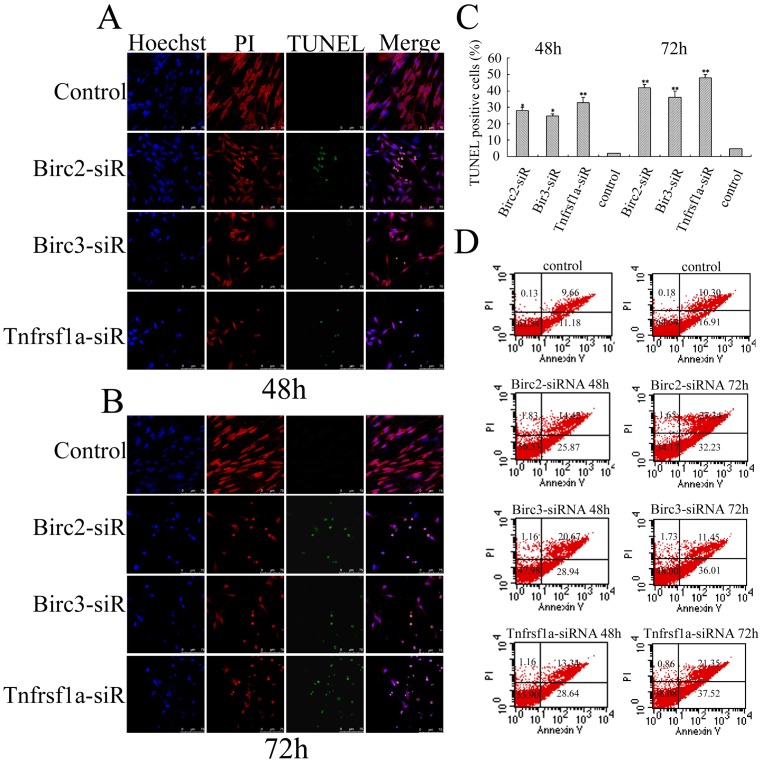
Apoptotic determination of SCs following siRNA knockdown. A and B, Confocal microscopy of TUNEL immunoreactivity after siRNA oligonucleotides transfection for 48 h and 72 h, respectively; C, Statistic analysis for A and B; D, Flow cytometric analysis of apoptotic SCs with a combination of Annexin V-FITC and Propidium Iodide after siRNA oligonucleotides transfection for 48 h and 72 h, respectively.

To understand whether Birc2, Birc3 and Tnfrsf1a inhibited apoptosis through caspase 3 and caspase 6 pathways, Birc2, Birc3 and Tnfrsf1a siRNA oligonucleotides were transfected in primary SCs with a purity of approximately 90% evaluated by S100 labeling (Figure 4D). The transfection efficiency measured by Cy3 control experiments is approximately 95% (Figure 4E). One of two siRNA oligonucleotides with higher efficiency of interference was selected for the following determination (Figure 4A–C). The expression of Birc2, Birc3 and Tnfrsf1a was specifically downregulated after transfection with corresponding siRNA at 48 h and 72 h, whereas the expression of control proteins was unaffected as revealed by immunoblotting (Figure 4A–C). The knockdown of Birc2, Birc3 or Tnfrsf1a resulted in an increased TUNEL-positive cell numbers (Figure 5A–C). To further confirm that siRNA oligonucleotides induce primary SCs apoptosis, cells were stained with annexin-V/propidium iodide (PI), and subsequently analyzed by flow cytometry. After siRNAs transfection for 48 h, a significant number of SCs showed annexin-V positive and PI negative staining, which increased from 11.18% of control cells to 25.87% for Birc2-siRNA, 28.94% for Birc2-siRNA and 28.64% for Tnfrsf1a-siRNA. The apoptotic SCs showed an augment after siRNAs transfection for 72 h, with the percentages increasing from 16.91% of control cells to 32.23% for Birc2-siRNA, 36.01% for Birc2-siRNA and 37.52% for Tnfrsf1a-siRNA (Figure 5D). The numbers of apoptotic cells measured by flow cytometry were less than those by the TUNEL method, due to the TUNEL's determination of the necrotic cells [31]. These results indicate that Birc2, Birc3 and Tnfrsf1a play roles of anti-apoptosis in SCs *in vitro*.

**Figure 6:**
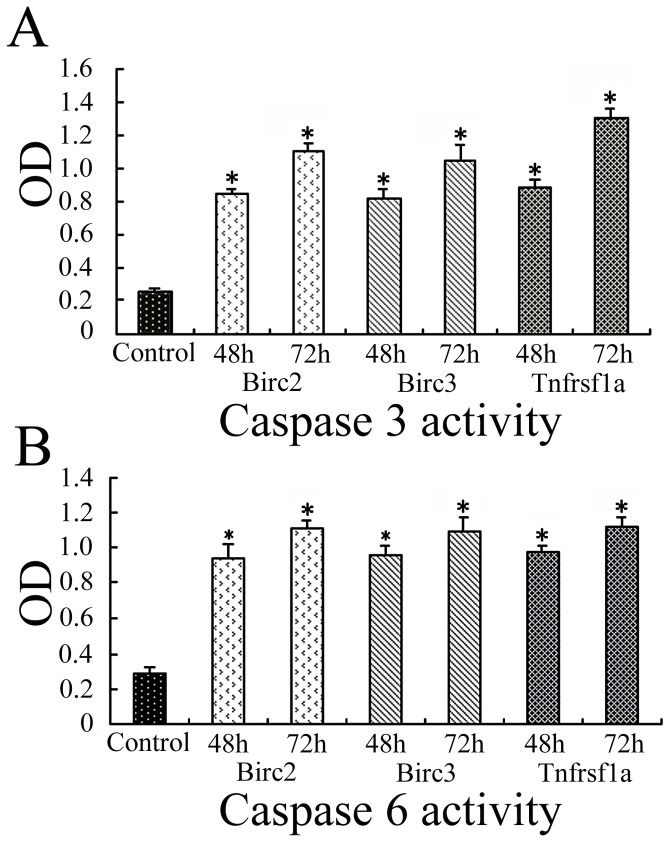
Caspase 3 and caspase 6 colorimetric assay. A, Showing the activities of caspase 3; B, Showing the activities of caspase 6. The enzymes activities in SCs after siRNA oligonucleotides transfection for 48 h and 72 h are measured by OD values from a specific colorimetric assay. Data are expressed as means ± SD of three independent experiments (n = 3). P<0.01.

The parallel experiments revealed that both caspase 3 and caspase 6 were upregulated in accordance with the knockdown of Birc2, Birc3 or Tnfrsf1a after transfection of siRNA at 48 h and 72 h, respectively (Figure 6A–B), indicating that the anti-apoptotic function of Birc2, Birc3 or Tnfrsf1a in SCs was performed by suppressing downstream target genes including caspase 3 and caspase 6. The results were entirely consistent with those of the gene network analysis.

## Discussion

A plethora of proofs reveal that TNF-α is able to potentiate SCs apoptosis through the modulation of their phenotype, and the distal segments of axotomised sciatic nerves are more susceptible to apoptosis [12]. These might be partly explained by analysis of gene network in current investigation showing that the apoptotic signaling pathway was triggered in several hours, and the anti-apoptotic protein Birc2 was not activated in the distal segments (Figure S1, S2). It has been regarded that adult SCs apoptosis is not a common phenomenon at early stages of peripheral nerves degeneration [32], but molecular changes do occur in the mature cells, including upregulation of p75^NTR^ expression [33], activation of c-Jun N-terminal kinase JNK and NFκB [34]. The levels of p75^NTR^ expression seem to be, to some extent, a precipitating factor to SCs apoptosis [35]. These suggested that, the inflammatory milieu elicited by TNF-α, have an effect on mature SCs physiology through apoptotic signal pathway. Interestingly, a high level of p75^NTR^ was also observed in the proximal nerve segments [12]. At meantime, signal transduction through anti-apoptotic molecules was also activated, implying that anti-apoptotic preservation of TNF-α-mediated pathway prevented the proximal nerves from the destruction of inflammatory milieu. It was noteworthy that the SCs from the distal segments also circumvented apoptosis. Whether Birc3 was involved in the TNF-α-mediated anti-apoptotic pathway, as it did in the proximal segments, remains further elucidated.

A dose more than 10 ng/ml of TNF-α potentiated the apoptosis of SCs *in vitro* [12], while less than the dose did not, as has previously been reported from several studies [36]. High dose of TNF-α promotes SCs apoptosis by increasing p75^NTR^ expression, and produced only a transient activation of NFκB [12]. However, a low dose of TNF-α was capable of partially restoring Schwann cell–axonal interactions in TNF^−/−^ cocultures by modulating downstream effectors [26], suggesting that TNF-α could regulate the fate of SCs in a dose-dependent manner through its activation of the TNFR1/complex 1 signal transduction pathway or other related death receptors such as p75^NTR^ known to induce SCs apoptosis [12].

TNFR1 ligation by TNF-α induces the formation of the receptor signaling complex, which employs the DD-containing adaptor molecule TRADD (TNF Receptor Associated Death Domain). Through its amino-terminal region and death domain TRADD in turn recruits TNFR-associated factor-2 (TRAF-2) and receptor interactive protein (RIP), which are required for the functional assembly of the TNFR1 signaling complex. By binding to TRAF2, Birc2 and Birc3 are recruited to TNFR-1 and -2 signaling complexes, where they modulate the activity of caspase 8, and then the activity of caspase 3, caspase 6, and caspase 7 [25], [37]. However, the exact molecular mechanism of anti-apoptotic activity of these two cellular Bircs, is still not well defined. Several studies revealed that the anti-apoptotic activity against various pro-apoptotic stimuli was mainly attributed to the other member of IAPs family, XIAP. While Birc2, Birc3 exhibited weak binding to and inhibition of caspases [37], [38]. Systematic level analysis following peripheral nerve injury demonstrated that Birc2 and Birc3 initiated the vital inhibition of caspases in response to the challenge of inflammation. XIAP, however, was not excavated from the gene network.

Expression levels of Tnfrsf1a were elevated after sciatic nerve tansection, and knockdown of the Tnfrsf1a in the primary cultured SCs resulted in the increased numbers of apoptosis, suggesting that Tnfrsf1a regulated the inflammation-mediated anti-apoptosis of peripheral nerves in canonical NFκB signaling pathways. The proximal stump is a heterogeneous tissue that includes connective tissue and microvasculature as well as macrophages or other cells of the immune system. Tissue immunohistochemistry demonstrated that Birc2, Birc3 and Tnfrsf1a positive staining colocalized with S100, indicating that it is SCs that were responsible for the inflammation-mediated anti-apoptosis of peripheral nerves.

In the same sub-network, the signaling pathway molecules of inflammatory cytokines IL-1 and IL-6 appeared as master regulators to mediate immune response (Figure 1, III). Il1rap and Il1r1 could interact with IL-1 to form the trimeric IL-1/Il1r1/Il1rap complex, thus triggers recruitment and binding of several intracellular adaptor proteins and kinases including Toll-interacting protein (Tollip), myeloid differentiation factor 88 (MyD88), and members of the IL-1R associated kinase (IRAK) family. These in turn activate downstream NFκB or the immune-related signaling pathway [39]. The PI3K/Akt/NFκB cascade could also be activated by IL-6, functions cooperatively to achieve the maximal anti-apoptotic effect of IL-6 against TGF-β. Therefore, NFκB is the key related regulators for inflammation-mediated signaling pathway. Birc2 and Birc3 are able to regulate pro-survival NFκB signaling [38], the two Bircs might be the most potential targets for anti-apoptotic protection mediated by inflammatory cytokines.

## Materials and Methods

### Animal surgery and tissue preparation

The experiments were performed on adult male Sprague-Dawley rats weighing 180–220 g. Thirty-six rats were randomly divided into six groups. The rats were anaesthetized by an intraperitoneal injection of complex narcotics (85 mg/kg trichloroac etaldehyde monohydrate, 42 mg/kg magnesium sulfate, 17 mg/kg sodium pentobarbital), and the sciatic nerve was exposed and lifted through an incision on the lateral aspect of the mid-thigh of left hind limb. A 1 cm long segment of sciatic nerve was then resected at the site just proximal to its division to tibial and common peroneal nerves, and the incision sites were then closed. The proximal segments of 0.5 cm were collected at 0 h, 0.5 h, 1 h, 3 h, 6 h and 9 h, and the distal segments of 0.5 cm were collected at 0 h, 0.5 h, 1 h, 3 h, 6 h, 9 h and 12 h after injury, respectively. The experiments were approved according to the Animal Care and Use Committee of Nantong University and the Jiangsu Province Animal Care Ethics Committee (Approval ID: SYXK(SU)2007-0021).

### cDNA array hybridization, data acquisition and evaluation

Total RNA was extracted using the mirVana™ miRNA Isolation Kit (Ambion, Austin, TX) according to the manufacturer's instructions. The quality of the purified RNA was assessed using a BioAnalyzer 2100 (Agilent Technology, Santa Clara, CA). The purified RNA was quantified by determining the absorbance at 260 nm using a Nanodrop ND-1000 spectrophotometer (Infinigen Biotechnology Inc., City of Industry, CA).

A cDNA array (Agilent Technology) was used from rat proximal nerve of 0 h, 0.5 h, 1 h, 3 h, 6 h and 9 h groups. The labeling and hybridization were performed at the Shanghai Biochip Company according to the protocols. Agilent Scan Control software was used for scanning the microarray slides, and Agilent Feature Extraction (FE) software version 9.5.3 was used for image analysis. Microarray data were analyzed using GeneSpring GX v11.0 software (Agilent Technology). All data is MIAME compliant, and the raw data has been deposited in a MIAME compliant database (NCBI Accession number: Series GSE33175), as detailed on the MGED Society website http://www.mged.org/Workgroups/MIAME/miame.html.

### Analysis of gene network

The RVM (Random variance model) F-test was applied to filter the differentially expressed genes of the control and experiment groups for raising degrees of freedom in the cases of small samples. After the significant analysis and FDR (false discovery rate) analysis, the differentially expressed genes were selected according to the p-value threshold [40]. The differential genes found at 6 time-points or 7 time-points were further undergone analysis for significant expression tendency (STC). Thus, STC and the genes belonging to it were thus obtained.

Based on the Gene Ontology (GO) database at NCBI, STC-GO analysis was applied to analyze the main function of the differential expression genes [29]. Generally, Fisher's exact test and χ^2^ test were used to classify the GO category, and the FDR was calculated to correct the P-value [41]. The FDR was defined as 
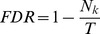
 (*N_k_* refers to the number of Fisher's test 7P-values less than those of χ^2^ test). Within the significant category, the enrichment Re was given by 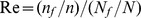, where *n_f_* is the number of differential genes within the particular category, *n* is the total number of genes within the same category, *N_f_* is the number of differential genes in the entire microarray, and *N* is the total number of genes in the microarray [42].

The pathway of the differential genes was analyzed according to the Kyoto Encyclopedia of Genes and Genomes (KEGG) bioinformatics database. Still, the Fisher's exact test and χ^2^ test were employed to select the significant ones, and the threshold was defined by P-value and FDR. The enrichment Re was calculated referring to the equation above [43]. A dynamic gene regulatory network was constructed according to gene fold expression and gene interaction in pathways. The relationships of the gene expression data were inferred using a continuous time recurrent neural network (CTRNN) as an abstract dynamic model for the gene regulatory network at six time-points, regardless of how such an interaction is realized in concrete biological terms [30]. The following is a general description of the CTRNN model: 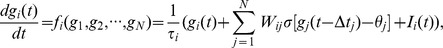 *τ_i_* denotes the time constant, *I_i_*(*t*) denotes the external input, *W_ij_* denotes gene *j* into gene *i*'s weight, *θ_j_* offset of gene *j*, 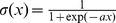 denotes sigmoid activation function, *g_i_*(*t*) denotes gene *i* expression value of the 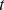 time point. The model parameters were estimated by using a genetic algorithm.

### TNF-α ELISA assay

Two-site sandwich ELISA was used to identify and quantify TNF-α in the extracts of 0.5 cm proximal segments collected at 0 h, 0.5 h, 1 h, 3 h, 6 h, 9 h and 12 h after injury, according to manufacturer's instructions (Duo-Set; R & D Systems, Minneapolis, MN).

### Real-time quantitative RT-PCR analysis

Quantitative real-time PCR amplification was carried out according to the manufacturer protocol of SYBR® PrimeScript™ RT-PCR Kit (Takara). Gene-specific primers were designed as follows: Tnfrsf1a-F: 5′-TGTTGCCTCTGGTTATCTT-3′, Tnfrsf1a-R: 5′-ACCCTCCACCTCTTTGAC-3′; Birc2-F: 5′-CTTAGTCAAGGGAAATGC-3′, Birc2-R: 5′-CAATGACAAGCCTGAAAC-3′; Birc3-F: 5′-TACTGTATTAGCGAAAGGAA-3′, Birc3-R: 5′-CAATGTCATCTGTGGGAAG-3′; β-actin-F: 5′-GTCACCAACTGGGACGAT-3′, β-actin-R: 5′-GAGGCATACAGGGACAACA-3′. Quantitative real-time PCR was performed with the 7300 real-time PCR system (Applied Biosystems). The relative expression value of each gene was determined using the manufacturer's 2^−ΔCt^ method and subsequent normalization to β-actin. Each sample was measured in duplicate. Data are provided as means ± SD.

### Schwann cells culture and treatment

SCs were prepared from sciatic nerves of adult male Sprague-Dawley rats and purified by complement lysis as described by Brockes et al. [27]. Cultures were expanded in Dulbecco's modified Eagle's medium (Gibco, Grand Island, NY, USA) supplemented with 10% (v/v) fetal calf serum. For apoptotic determination, SCs with 90% confluency were treated with 0–40 ng/ml soluble recombinant rat TNF-α (Peprotech, endotoxin level <1 EU/µg) for 12 h and 24 h, respectively. SCs with 30–50% confluency were transfected with 40 nM siRNA using Lipofectamine™ RNAiMAX transfection kit (Invitrogen) according to the protocol. Cells at 24 h, 48 h and 72 h were collected and stored at −20°C for biochemical assay, or fixed and immunolabeled for S100 and Hoechst to assess the total number of SCs. Fluorescently tagged control Cy3 was used as a marker for transfection.

### Western blot

Protein was extracted from SCs, proximal and distal segments with a buffer containing 1% SDS, 100 mM Tris–HCl, 1 mM PMSF, and 0.1 mM β-mercaptoethanol. Protein concentration of each specimen was detected by the Bradford method to maintain the same loads. Protein extracts were heat denatured at 95°C for 5 min, electrophoretically separated on 10% SDS–PAGE, and transferred to PVDF membranes. The membranes were subjected to the reaction with a 1∶1000 dilution of cleaved caspase 3 (Asp175, Cell Signaling), 1∶1000 dilution of monoclonal anti-Bcl2 antibody (Sigma), 1∶1000 dilution of monoclonal anti-Bax antibody (Sigma), 1∶1000 dilution of goat anti-Birc2 polyclonal antibody, 1∶1000 dilution of rabbit anti-Birc3 polyclonal antibody (Santa Cruz, CA) or 1∶1000 dilution of rabbit anti-Tnfrsf1a polyclonal antibody (Abcam, Hongkong) in TBS buffer at 4°C overnight, followed by a reaction with secondary antibody conjugated with horseradish peroxidase (HRP) (donkey anti-goat HRP dilution 1∶2000 or goat anti-rabbit HRP dilution 1∶1000, Santa Cruz) at room temperature for 2 h. After the membrane was washed, the HRP activity was detected using an ECL kit. The image was scanned with a GS800 Densitometer Scanner (Bio-Rad), and the data were analyzed using PDQuest 7.2.0 software (Bio-Rad). β-actin (1∶5000) was used as an internal control.

### Tissue immunohistochemistry

The sciatic nerve was harvested, post-fixed and sectioned. Sections were allowed to incubate with goat anti-Birc2 polyclonal antibody (1∶50 dilution, Santa Cruz, CA), rabbit anti-Birc3 polyclonal antibody (1∶100 dilution, Santa Cruz, CA), rabbit anti-Tnfrsf1a polyclonal antibody (Abcam, Hongkong), and rabbit anti-S100 polyclonal antibody (1∶400 dilution, Sigma) or mouse anti- S100 monoclonal antibody (1∶400 dilution, Sigma) at 4°C for 24 h. The sections were further reacted with the FITC-labeled secondary antibody goat anti-mouse IgG (1∶200 dilution, Gibco), the Cy3-labeled secondary antibody goat anti-rabbit IgG (1∶200 dilution, Santa Cruz), the FITC-labeled secondary antibody donkey anti-goat IgG (1∶200 dilution, Gibco), or the TRITC-labeled secondary antibody donkey anti-rabbit IgG (1∶200 dilution, Gibco) at 4°C overnight, followed by observation under a confocal laser scanning microscope (Leica, Heidelberg, Germany).

### TdT-Mediated dUTP-Biotin Nick end Labeling (TUNEL) assay

The section and the cells after siRNA transfection and fixing in 4.0% paraformaldehyde for 20 min,were subjected to TUNEL assay by the Dead EndTM Fluorometric TUNEL system (Promega, Madison, WI) according to the manufacturer's specifications. Cells were then incubated simultaneously with 5 µM Hoechst 33342 (staining healthy cells) and 5 µg/ml propidium iodide (Sigma), for 15 min in a water bath at 37°C. Reaction was visualized under a TCS SP2 confocal microscope and DMR fluorescence microscope (Leica Microsystems, Wetzlar, Germany). Three independent experiments were performed in duplicate and at least 30 fields from each independent experiment were imaged using confocal microscopy.

### Flow cytometry

The SCs were harvested and resuspended in a 1× binding buffer (10 mM HEPES, 140 mmM NaCl, 2.5 mM CaCl_2_) at a concentration of 1×10^6^ cell/ml. In addition to a 100 µl aliquot of the cell suspension, 5 µl of FITC-conjugated annexin V (PharMingen, San Diego, CA) and 5 µl of 50 µg/ml propidium iodide (PI) were added. After incubation for 15 min in the dark at room temperature, cells were analyzed for annexin V binding within 1 h with a flow cytometer (BD FACScalibur, BD Bioscience, San Jose, CA).

### Caspase 3 and caspase 6 colorimetric assays

The SCs cells were collected and washed with phosphate buffer saline (PBS, pH 7.2). After centrifugation, caspase 3 and caspase 6 colorimetric assays were performed with an EIX-800 Microelisa reader (Bio-Tek Inc., USA) according to the manufacturer's specifications (Abcam).

## Supporting Information

Figure S1 **Gene network analysis of the distal nerve segments in short time-series following rat sciatic nerve transection.** The lines indicate the interactions between each gene, and the weight value is illustrated by the width of lines.
(TIF)

Figure S2 **The comparative analysis of Birc2, Birc3 and Tnfrsf1a protein levels in the proximal and distal segments, and apoptotic detection of distal segments.** A, Western blot of Birc2, Birc3 and Tnfrsf1a in the proximal segments; B, Western blot of Birc2, Birc3, Tnfrsf1a and cleaved caspase 3 in the distal segments; C, TUNEL staining of the distal segments at 0 h, 1 h, 6 h and 12 h after rat sciatic nerve transection.
(TIF)

Table S1 **KEGG pathway analysis of distal segment.**
(DOC)

Table S2 **Effect of key regulated gene on the downstream genes.**
(DOC)
