# Supplementary figures and images for: Gene Network Revealed Involvements of Birc2, Birc3 and Tnfrsf1a in Anti-Apoptosis of Injured Peripheral Nerves
Source: PLoS One. 2012 Sep 17;7(9):e43436. doi: 10.1371/journal.pone.0043436 (PMC3444457; doi:10.1371/journal.pone.0043436)

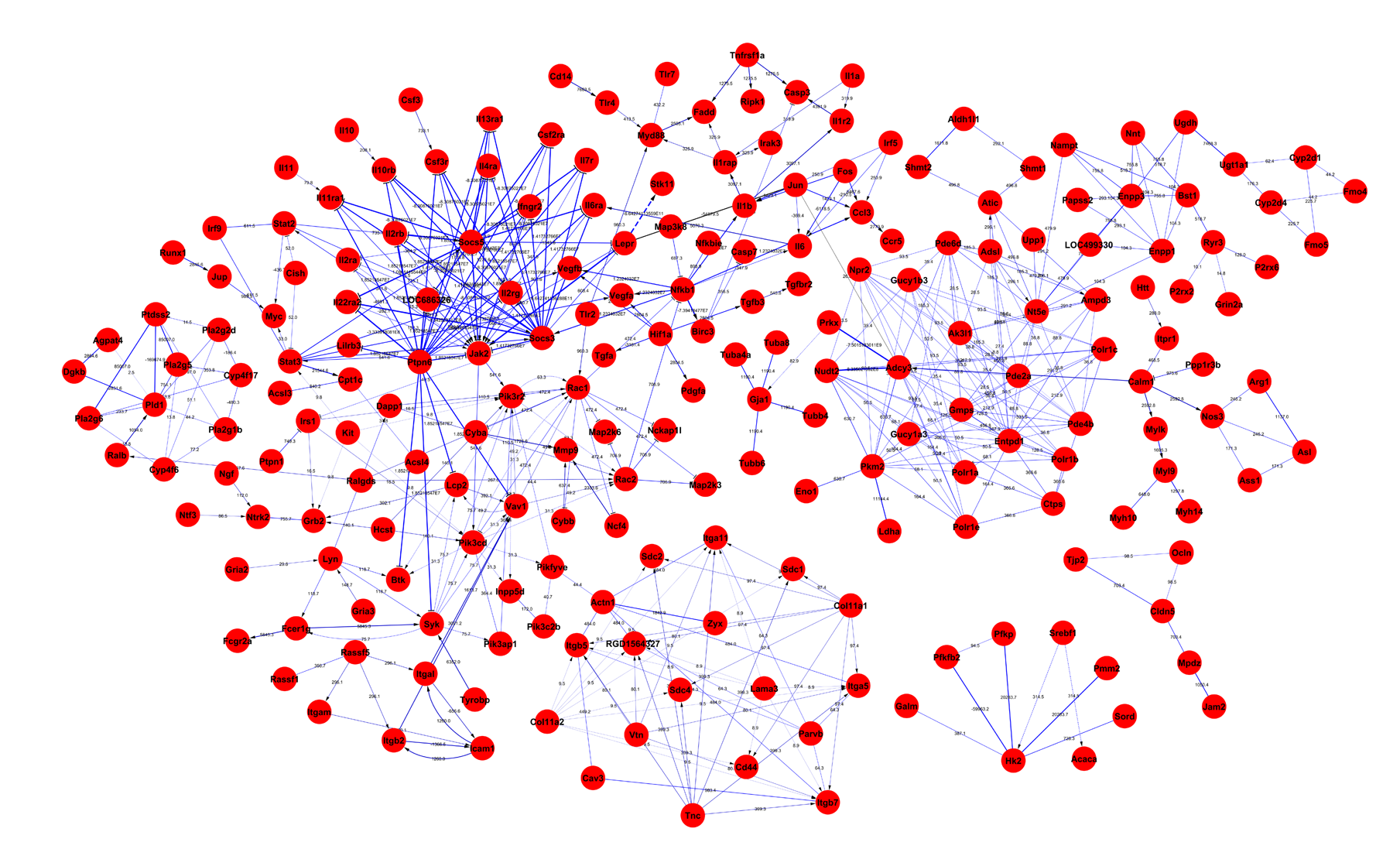

Supplement: Figure S1 — Gene network analysis of the distal nerve segments in short time-series following rat sciatic nerve transection. The lines indicate the interactions between each gene, and the weight value is illustrated by the width of lines. (TIF) [file pone.0043436.s001.tif]

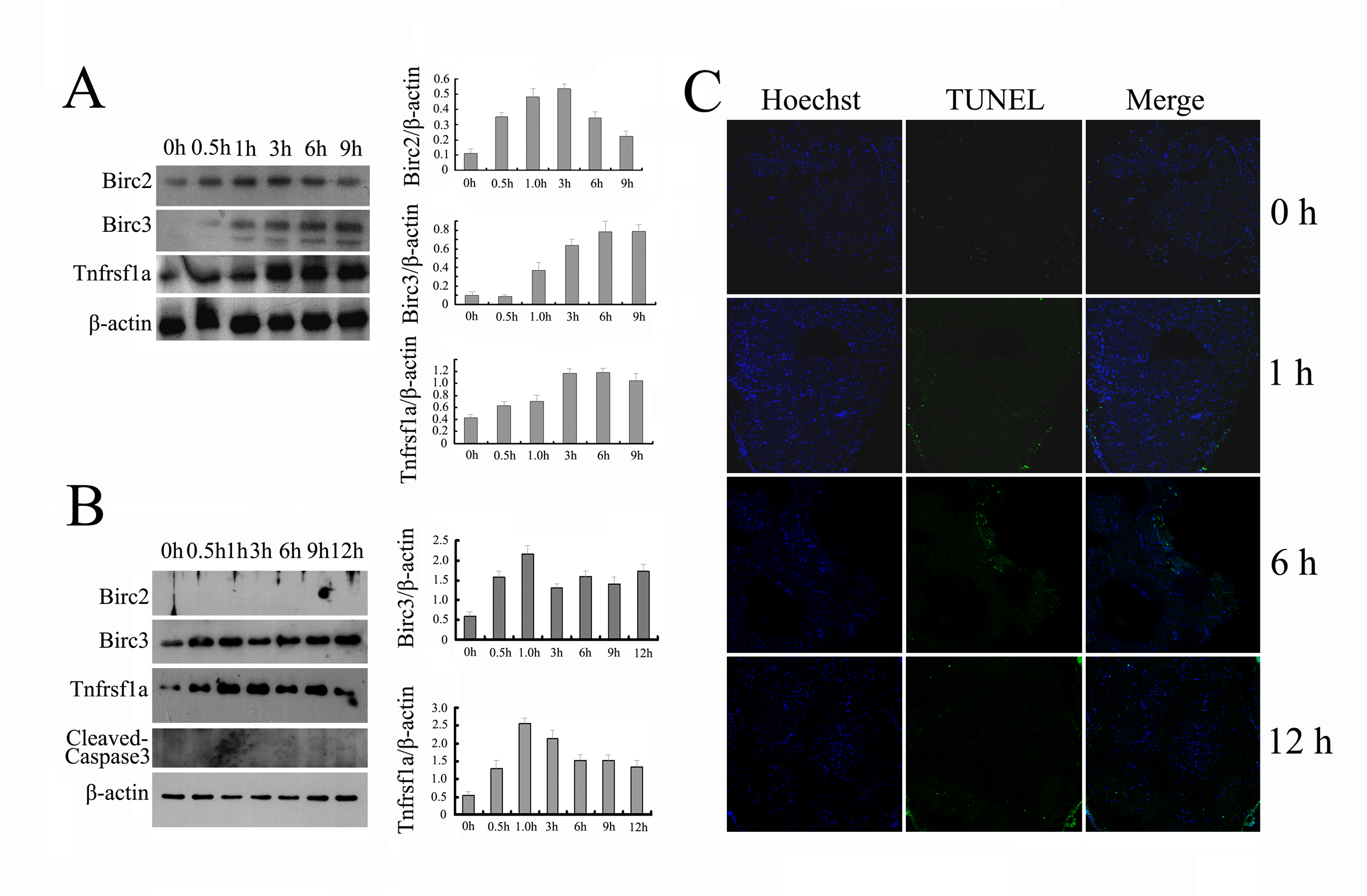

Supplement: Figure S2 — The comparative analysis of Birc2, Birc3 and Tnfrsf1a protein levels in the proximal and distal segments, and apoptotic detection of distal segments. A, Western blot of Birc2, Birc3 and Tnfrsf1a in the proximal segments; B, Western blot of Birc2, Birc3, Tnfrsf1a and cleaved caspase 3 in the distal segments; C, TUNEL staining of the distal segments at 0 h, 1 h, 6 h and 12 h after rat sciatic nerve transection. (TIF) [file pone.0043436.s002.tif]
