# Supplementary material for: Gene Network Revealed Involvements of Birc2, Birc3 and Tnfrsf1a in Anti-Apoptosis of Injured Peripheral Nerves
Source: PLoS One. 2012 Sep 17;7(9):e43436. doi: 10.1371/journal.pone.0043436 (PMC3444457; doi:10.1371/journal.pone.0043436)
Supplement: Table S1 — KEGG pathway analysis of distal segment. (DOC) [file pone.0043436.s003.doc]

| Pathway | Count | *P* value | qFDR |
| --- | --- | --- | --- |
| Cytokine-cytokine receptor interaction | 50 | 3.54E-21 | 1.41E-19 |
| MAPK signaling pathway | 52 | 2.47E-18 | 4.91E-17 |
| Hematopoietic cell lineage | 25 | 1.42E-14 | 1.88E-13 |
| Jak-STAT signaling pathway | 33 | 1.49E-13 | 1.48E-12 |
| Olfactory transduction | 3 | 2.49E-12 | 1.98E-11 |
| Cell adhesion molecules (CAMs) | 30 | 8.95E-11 | 5.53E-10 |
| Toll-like receptor signaling pathway | 23 | 1.85E-10 | 9.17E-10 |
| Purine metabolism | 25 | 8.00E-09 | 3.29E-08 |
| Fc epsilon RI signaling pathway | 18 | 9.61E-09 | 3.82E-08 |
| B cell receptor signaling pathway | 18 | 1.54E-08 | 5.56E-08 |
| Nicotinate and nicotinamide metabolism | 8 | 5.22E-08 | 1.61E-07 |
| Natural killer cell mediated cytotoxicity | 21 | 5.44E-08 | 1.66E-07 |
| Aminoacyl-tRNA biosynthesis | 12 | 7.47E-08 | 2.12E-07 |
| Regulation of actin cytoskeleton | 28 | 1.24E-06 | 2.90E-06 |
| Arginine and proline metabolism | 10 | 1.40E-06 | 3.20E-06 |
| Leukocyte transendothelial migration | 20 | 1.85E-06 | 3.95E-06 |
| Apoptosis | 18 | 2.01E-06 | 4.21E-06 |
| Focal adhesion | 26 | 3.33E-06 | 6.62E-06 |
| Neuroactive ligand-receptor interaction | 31 | 5.48E-06 | 1.03E-05 |
| Glycerophospholipid metabolism | 11 | 7.43E-06 | 1.34E-05 |
| Adipocytokine signaling pathway | 13 | 2.27E-05 | 3.76E-05 |
| VEGF signaling pathway | 13 | 6.05E-05 | 9.50E-05 |
| ECM-receptor interaction | 13 | 8.17E-05 | 0.0001249 |
| Insulin signaling pathway | 18 | 0.0001594 | 0.000224 |
| Gap junction | 14 | 0.0001685 | 0.0002346 |
| T cell receptor signaling pathway | 15 | 0.0001846 | 0.0002529 |
| Proteasome | 7 | 0.0002402 | 0.0003181 |
| Arachidonic acid metabolism | 10 | 0.0005401 | 0.0006702 |
| GnRH signaling pathway | 13 | 0.0005683 | 0.0007011 |
| Primary immunodeficiency | 8 | 0.0006859 | 0.0008258 |
