# Supplementary material for: Gene Network Revealed Involvements of Birc2, Birc3 and Tnfrsf1a in Anti-Apoptosis of Injured Peripheral Nerves
Source: PLoS One. 2012 Sep 17;7(9):e43436. doi: 10.1371/journal.pone.0043436 (PMC3444457; doi:10.1371/journal.pone.0043436)
Supplement: Table S2 — Effect of key regulated gene on the downstream genes. (DOC) [file pone.0043436.s004.doc]

| gene1 | gene2 | weight value | interaction |
| --- | --- | --- | --- |
| Lepr | Jak2 | 6.64E+11 | activation(phosphorylation) |
| Lepr | Stk11 | -6.64E+11 | activation(indirect effect) |
| Adcy3 | Prkx | -7500000000 | compound |
| Socs5 | Il22ra2 | 1080000000 | inhibition(ubiquination) |
| Adcy3 | Nudt2 | 8.34E+08 | compound |
| Ptpn6 | Il22ra2 | -333000000 | inhibition |
| Socs3 | Il22ra2 | -184000000 | inhibition(ubiquination) |
| Socs5 | LOC686326 | -8.31E+07 | inhibition(ubiquination) |
| Socs5 | Jak2 | -8.31E+07 | inhibition(ubiquination) |
| Socs5 | Il7r | -8.31E+07 | inhibition(ubiquination) |
| Socs5 | Il6ra | -8.31E+07 | inhibition(ubiquination) |
| Socs5 | Il4ra | -8.31E+07 | inhibition(ubiquination) |
| Socs5 | Il2rg | -8.31E+07 | inhibition(ubiquination) |
| Socs5 | Il2rb | -8.31E+07 | inhibition(ubiquination) |
| Socs5 | Il13ra1 | -8.31E+07 | inhibition(ubiquination) |
| Socs5 | Il11ra1 | -8.31E+07 | inhibition(ubiquination) |
| Socs5 | Il10rb | -8.31E+07 | inhibition(ubiquination) |
| Socs5 | Ifngr2 | -8.31E+07 | inhibition(ubiquination) |
| Socs5 | Csf2ra | -8.31E+07 | inhibition(ubiquination) |
| Socs5 | Csf3r | -8.31E+07 | inhibition(ubiquination) |
| Nfkb1 | Birc3 | -7.39E+07 | expression |
| Ptpn6 | Lilrb3 | 1.85E+07 | binding/association |
| Ptpn6 | Csf3r | 1.85E+07 | inhibition |
| Ptpn6 | LOC686326 | 1.85E+07 | inhibition |
| Ptpn6 | Jak2 | 1.85E+07 | inhibition |
| Ptpn6 | Il7r | 1.85E+07 | inhibition |
| Ptpn6 | Il6ra | 1.85E+07 | inhibition |
| Ptpn6 | Il4ra | 1.85E+07 | inhibition |
| Ptpn6 | Il2rg | 1.85E+07 | inhibition |
| Ptpn6 | Il2rb | 1.85E+07 | inhibition |
| Ptpn6 | Il13ra1 | 1.85E+07 | inhibition |
| Ptpn6 | Il11ra1 | 1.85E+07 | inhibition |
| Ptpn6 | Il10rb | 1.85E+07 | inhibition |
| Ptpn6 | Ifngr2 | 1.85E+07 | inhibition |
| Ptpn6 | Csf2ra | 1.85E+07 | inhibition |
| Ptpn6 | Lcp2 | 1.85E+07 | inhibition(dephosphorylation) |
| Ptpn6 | Vav1 | 1.85E+07 | inhibition(dephosphorylation) |
| Ptpn6 | Syk | 1.85E+07 | inhibition(dephosphorylation) |
| Ptpn6 | Btk | 1.85E+07 | inhibition(dephosphorylation) |
| Socs3 | Csf2ra | 1.42E+07 | inhibition(ubiquination) |
| Socs3 | LOC686326 | 1.42E+07 | inhibition(ubiquination) |
| Socs3 | Jak2 | 1.42E+07 | inhibition(ubiquination) |
| Socs3 | Il7r | 1.42E+07 | inhibition(ubiquination) |
| Socs3 | Il6ra | 1.42E+07 | inhibition(ubiquination) |
| Socs3 | Il4ra | 1.42E+07 | inhibition(ubiquination) |
| Socs3 | Il2rg | 1.42E+07 | inhibition(ubiquination) |
| Socs3 | Il2rb | 1.42E+07 | inhibition(ubiquination) |
| Socs3 | Il13ra1 | 1.42E+07 | inhibition(ubiquination) |
| Socs3 | Il11ra1 | 1.42E+07 | inhibition(ubiquination) |
| Socs3 | Il10rb | 1.42E+07 | inhibition(ubiquination) |
| Socs3 | Ifngr2 | 1.42E+07 | inhibition(ubiquination) |
| Socs3 | Csf3r | 1.42E+07 | inhibition(ubiquination) |
| Nfkb1 | Il1b | 1.23E+07 | expression |
| Nfkb1 | Ccl3 | 1.23E+07 | expression |
| Nfkb1 | Vegfb | 1.23E+07 | expression |
| Nfkb1 | Vegfa | 1.23E+07 | expression |
| Nfkb1 | Socs3 | 1.23E+07 | expression |
| Ptdss2 | Pld1 | -1.70E+05 | compound |
| Ptdss2 | Pla2g5 | 8.50E+04 | compound |
| Ptdss2 | Pla2g6 | 8.50E+04 | compound |
| Hk2 | Pfkfb2 | -6.00E+04 | compound |
| Lepr | Jun | -5.49E+04 |  |
| Stat3 | Cpt1c | 2.15E+04 | expression |
| Hk2 | Pfkp | 2.03E+04 | compound |
| Hk2 | Pmm2 | 2.03E+04 | compound |
| Ldha | Pkm2 | 1.11E+04 | compound |
| Cd14 | Tlr4 | 7.66E+03 | activation |
| Ugdh | Ugt1a1 | 7.47E+03 | compound |
| Tyrobp | Syk | 6.35E+03 | activation |
| Fos | Il6 | -6.12E+03 | expression |
| Fcer1g | Syk | 5.85E+03 | activation |
| Fcer1g | Fcgr2a | 5.85E+03 | binding/association |
| Fos | Il1b | 5.47E+03 | expression |
| Fos | Ccl3 | 5.47E+03 | expression |
| Il6 | Il6ra | 5.07E+03 | activation |
| Stat3 | Vegfb | -4.89E+03 | expression |
| Stat3 | Vegfa | -4.89E+03 | expression |
| Stat3 | Socs5 | -4.89E+03 | expression |
| Stat3 | Socs3 | -4.89E+03 | expression |
| Il1r2 | Casp3 | 4.39E+03 | activation |
| Hif1a | Tgfa | -3.16E+03 | expression |
| Il1b | Il1rap | 3.10E+03 | activation |
| Il1b | Il1r2 | 3.10E+03 | activation |
| Itgb2 | Vav1 | 3.00E+03 | activation |
| Dgkb | Agpat4 | 2.86E+03 | compound |
| Runx1 | Jup | 2.85E+03 | expression |
| Dgkb | Pld1 | -2.83E+03 | compound |
| Hif1a | Vegfb | 2.80E+03 | expression |
| Hif1a | Vegfa | 2.80E+03 | expression |
| Hif1a | Tgfb3 | 2.80E+03 | expression |
| Hif1a | Pdgfa | 2.80E+03 | expression |
| Ccl3 | Ccr5 | 2.78E+03 | activation |
| Calm1 | Mylk | 2.59E+03 | activation |
| Calm1 | Adcy3 | 2.59E+03 | activation |
| Calm1 | Nos3 | 2.59E+03 | binding/association |
| Myd88 | Fadd | 2.51E+03 | activation |
| Ncf4 | Mmp9 | 2.33E+03 | activation |
| Zyx | Actn1 | 1.84E+03 | binding/association |
| Cyba | Mmp9 | 1.73E+03 | activation |
| Csf3r | Jak2 | 1.71E+03 | phosphorylation |
| Mylk | Myl9 | 1.70E+03 | activation(phosphorylation) |
| Ifngr2 | Jak2 | 1.62E+03 | phosphorylation |
| Itgal | Vav1 | 1.61E+03 | activation |
| Shmt2 | Aldh1l1 | 1.61E+03 | compound |
| Jun | Il1b | 1.42E+03 | expression |
| Jun | Ccl3 | 1.42E+03 | expression |
| Itgb2 | Icam1 | -1.37E+03 | activation |
| Socs5 | Lepr | -1.34E+03 | inhibition(ubiquination) |
| Myh14 | Myl9 | 1.30E+03 | binding/association |
| Il4ra | Jak2 | 1.28E+03 | phosphorylation |
| Tnfrsf1a | Fadd | 1.28E+03 | activation |
| Tnfrsf1a | Casp3 | 1.28E+03 | activation |
| Tnfrsf1a | Ripk1 | 1.28E+03 | activation |
| Icam1 | Itgb2 | 1.26E+03 | activation |
| Icam1 | Itgal | 1.26E+03 | activation |
| Il10rb | Jak2 | 1.25E+03 | phosphorylation |
| Gja1 | Tuba4a | 1.19E+03 | binding/association |
| Gja1 | Tuba8 | 1.19E+03 | binding/association |
| Gja1 | Tubb4 | 1.19E+03 | binding/association |
| Gja1 | Tubb6 | 1.19E+03 | binding/association |
| Arg1 | Asl | 1.14E+03 | compound |
| Ralb | Pld1 | 1.05E+03 | activation |
| Jam2 | Mpdz | 1.05E+03 | binding/association |
